# Chemokine profiles of interstitial pneumonia in patients with dermatomyositis: a case control study

**DOI:** 10.1038/s41598-017-01685-5

**Published:** 2017-05-09

**Authors:** Katsuhiro Oda, Takuya Kotani, Tohru Takeuchi, Takaaki Ishida, Takeshi Shoda, Kentaro Isoda, Shuzo Yoshida, Yasuichiro Nishimura, Shigeki Makino

**Affiliations:** 1 Department of Internal Medicine (I), Osaka Medical College, Takatsuki, Osaka Japan; 2 Department of Mathematics, Osaka Medical College, Takatsuki, Osaka Japan

## Abstract

Chemokines play an important role in the pathophysiology of dermatomyositis (DM) with interstitial pneumonia (IP). However, the relation between chemokines and the disease activity or prognosis of DM-IP has not been elucidated. We evaluated the serum C-C motif chemokine ligand (CCL) 2, Th1 chemokines (C-X-C motif chemokine ligand [CXCL] 9, CXCL10, CXCL11), and Th2 chemokine (CCL17) profiles of 30 patients, and examined the relation between these chemokines and the disease activity or prognosis of DM-IP. Initial serum CCL2 level was higher in the death group (*P* = 0.007). To determine the cut-off points effective as poor prognostic factors of DM-IP, ROC curve analysis was carried out on initial serum CCL2 level. The value that maximized the area under the ROC curve was 894 pg/mL (sensitivity: 100%, specificity: 70.8%). Serum CCL2, CXCL9, CXCL10, and CXCL11 levels were lower at 2 weeks after treatment initiation than before treatment. Serum CCL2, CXCL10, and CXCL11 levels at 2 weeks after treatment initiation were higher in the death group. Serum levels of chemokines such as CCL2, CXCL10, and CXCL11 may be possible biomarkers of disease activity and prognosis in DM-IP, and serum CCL2 level may be useful when deciding initial treatment.

## Introduction

Dermatomyositis (DM) is one of the idiopathic inflammatory myopathies characterized by inflammation of skin and muscle^1, 2^. Clinically amyopathic dermatomyositis (CADM) is a subgroup of DM characterized by typical skin rash and little or no evidence of myositis^3, 4^. DM is frequently complicated with interstitial pneumonia (IP), causing increased morbidity and mortality^5, 6^. Based on the disease progression of IP, DM-IP is classified into two groups: DM with acute/subacute IP (A/SIP) and DM with chronic IP (CIP). Patients with DM-CIP are treated with prednisolone (PDN) alone or the combination of PDN and calcineurin inhibitors or azathioprine with favorable response. In DM-A/SIP, early intervention with the combination of PDN and calcineurin inhibitors such as cyclosporin-A (CSA) or tacrolimus (TAC) and/or intravenous cyclophosphamide pulse therapy (IVCY) and/or intravenous immunoglobulin improves the therapeutic prognosis^7–13^. However, some patients are resistant to these combination therapies and die. Thus, in determining the appropriate treatment, it is clinically important to find factors that can predict the progress and prognosis of DM-IP.

Anti-aminoacyl-tRNA synthetase (ARS) antibody has been found to be a specific autoantibody for polymyositis (PM) and DM and is strongest with IP^14^. Eight anti-ARS antibodies including anti-Jo-1 antibody have been identified. Anti-ARS antibody-positive PM/DM-IP shows a good response to immunosuppressive therapy with a good prognosis^15^. Sato *et al*. detected anti-melanoma differentiation-associated gene 5 (MDA5) antibodies in a high percentage of CADM patients and reported that about half of patients with anti-MDA5 antibody-positive CADM had A/SIP^16^. Gono *et al*. showed that the prognosis was significantly poorer in DM-IP patients with anti-MDA5 antibody than anti-ARS antibody^17^.

Previous reports revealed that several prognostic factors, including positive anti-MDA5 antibody, high serum ferritin level before treatment, high alveolar-arterial oxygen difference (AaDO_2_), low % forced vital capacity, and high total ground-glass opacity (GGO) score before treatment, and elevation of Krebs von den Lungen-6 (KL-6) at 2–4 weeks after the beginning of treatment, were poor prognostic factors of DM-IP^16–25^. Gono *et al*. reported that the survival rate of DM-IP patients was significantly lower with a serum ferritin of ≥1,500 ng/mL^18^. They also reported that among anti-MDA5 antibody-positive DM-IP the survival rate was significantly lower with a serum ferritin level of ≥500 ng/mL and all patients with a serum ferritin level of ≥1,600 ng/mL died^17^. Isoda *et al*. reported that the survival rate was lower with a serum ferritin level of ≥600 ng/mL and AaDO_2_ ≥ 45 mmHg^21^. Kang *et al*. reported that initial % forced vital capacity ≤60% was associated with poor prognosis in patients with PM/DM-IP^22^. The relationship between the CT score quantifying the whole lung and outcomes in DM-IP patients has already been investigated and it was reported that the total CT score was significantly higher in the patients who died from rather than survived DM-IP^23, 24^.

The association between serum cytokine levels before treatment and disease activity or prognosis of DM-IP was examined. Interferon-α, tumor necrosis factor-α, interleukin (IL)-6, IL-10, and IL-18 were related to disease activity^20, 26–32^. Patients with a high serum IL-6 level had a poor prognosis^29^. Chemokines induce directed chemotaxis and activation of various leukocytes and relate to the pathophysiological conditions of several diseases including DM-IP. C-C motif chemokine ligand (CCL) 2, CCL17, C-X-C motif chemokine ligand (CXCL) 8, and CXCL10 levels were higher in DM patients with IP than in those without IP^28, 30, 33, 34^. Gono *et al*. reported that high serum CXCL8 level was a poor prognostic factor of DM-IP^28^. The relation between these chemokines and the disease activity or prognosis of DM-IP has not been elucidated.

We evaluated serum chemokines before and after treatment and examined the relation between serum chemokines and the disease activity or prognosis of DM-IP. We also examined the relation between serum chemokines and the previously reported biomarkers.

## Methods

### Patients

We examined patients who were admitted to Osaka Medical College Hospital from October 2011 to March 2015 in this retrospective study. They were diagnosed as having DM or CADM based on the criteria of Bohan and Peter^1, 2^ or Sontheimer and Gerami *et al*.^3, 4^. Patients with other connective tissue diseases and malignancies were excluded. IP was diagnosed with chest high-resolution computed tomography (HRCT). A/SIP was defined as IP in which the respiratory condition, laboratory findings, arterial blood gas findings, chest HRCT images, and pulmonary function test findings aggravated rapidly within 3 months^35^. CIP did not fulfill the definition of A/SIP. Clinical data were obtained from medical records on admission. This study was conducted in accordance with the Declaration of Helsinki and its amendments and was approved by Osaka Medical College and the Faculties of Medicine Ethics Committee (approval nos 1316, 1366). Informed consent was obtained from each patient.

### Treatments

PDN (0.5–1.0 mg/kg/day) was administered in all patients. CSA or TAC was used as combination treatment according to the decision of the physician. CSA was started at 4 mg/kg/day once a day before breakfast, and the concentration at 2 hours after administration was adjusted to 1500 ng/mL or above. TAC was started at 0.1 mg/kg/day twice a day before breakfast and dinner, and the trough was adjusted to 15–20 ng/mL. Whether additional treatments such as methylprednisolone pulse therapy, IVCY, or intravenous immunoglobulin were administered was determined by the physicians depending on each patient’s condition.

### Measurement of laboratory parameters

The laboratory parameters measured were creatine kinase (CK), aldolase (ALD), lactic acid dehydrogenase (LDH), C-reactive protein (CRP), KL-6, and ferritin. Blood serum samples were collected on admission and at 2 weeks after treatment initiation. Serum samples were stored at −70 °C until measurement.

Serum values of chemokines were assayed with a cytometric bead array method (BD Bioscience, San Jose, CA, USA). Detection limits of chemokines CCL2, CCL17, CXCL9, CXCL10, and CXCL11 were 43.5, 93.92, 125.3, 3.54, and 2.19 pg/ml, respectively.

Anti-MDA5 antibody was determined by ELISA using recombinant MDA5 antigen (OriGene, Rockville, MD, USA) as described previously^36^. Anti-ARS antibody was determined using a commercially available line blot test kit (Myositis Profile Euroline Blot test kit; Euroimmun, Lübeck, Germany).

### Arterial blood gas analysis and pulmonary function test

Arterial blood gas analysis including PaO_2_, PaCO_2_, and PaO_2_/FiO_2_ (P/F) ratio was carried out on admission. Respiratory function was measured by spirometry (SYSTEM21; Minato Medical Science, Osaka, Japan). Vital capacity (VC) was determined by the N2 washout method, and diffusion capacity of the lung for carbon monoxide (DLco) was determined by the single-breath method^37–39^. Respiratory function test results are expressed as percentages of the predicted value.

### HRCT scoring

HRCT was performed using a 64-detector row CT Aquilon multiscanner (Toshiba Medical Systems Corporation, Tokyo, Japan). Slice thickness was 1.0–1.5 mm every 10 mm, with the scan area including the entire lung. All patients underwent chest HRCT prior to treatment, and images were reviewed independently by 3 observers (TK, TI, and TS) blinded to the patients’ clinical information. Inter-observer disagreements were resolved by consensus. GGO and fibrosis were both scored to assess HRCT findings, as previously described^40^. Each patient’s lobe was scored by the same observers, and the average value was used. The scores obtained were summed as the total CT score.

### Statistical analysis

Fisher’s exact test was used when appropriate, and the Mann-Whitney U-test was used for the comparison of median values. Data are presented as the median (interquartile range). The multivariate analysis was performed using logistic regression analysis. Wilcoxon’s rank sum test was used to assess the chemokine levels initially and at 2 weeks after treatment initiation. We used receiver operating characteristic (ROC) curve analysis to determine the most suitable cut-off level for prediction of the prognosis of DM-IP. We used the Kaplan-Meier method to assess survival curves and the log-rank test to evaluate the significance of differences between the two groups. Correlations were evaluated using Spearman’s correlation coefficients. A *P* value of <0.05 was considered to indicate significance. The data were analyzed using JMP version 12.0 (SAS Institute Inc., Cary, NC, USA).

## Results

### Clinical characteristics, chemokine levels, and contents of treatment between survivors and non-survivors

Of the 35 DM-IP patients, 24 survived and 11 died. Of the 11 dead patients, 6 died due to the exacerbation of DM-IP within 12 weeks after the beginning of treatment. Of the remaining 5 patients, 2 died due to sepsis, 1 due to cytomegalovirus infection, 1 due to pulmonary aspergillosis, and 1 due to thrombotic thrombocytopenic purpura. The subjects included 22A/SIP and 8 CIP patients. Four of the 22A/SIP patients were initially diagnosed with CIP. However, the disease type was changed to A/SIP because of the rapid progression of IP. Therefore, the patients underwent therapeutic intervention. Also, all the CIP patients underwent therapeutic intervention because of the gradual progression of IP for more than 4 months to several years. We compared the clinical and laboratory findings between the 24 survivors and 6 patients who died due to IP exacerbation to reveal biomarkers for disease activity and prognosis in DM-IP. Table 1 shows the clinical findings and contents of treatment in the 24 survivors and 6 dead patients. The following data are reported as the median level (range). No significant differences were observed in age, sex, frequency of CADM and A/SIP, and time from the appearance of respiratory symptoms to treatment initiation. The anti-MDA5 antibody-positive rate was higher in the death group (67%) than in the survival group (17%) (*P* = 0.029). There were no significant differences in the anti-ARS antibody-positive rate and in CK, ALD, LDH, CRP, KL-6, and ferritin levels between the two groups. However, KL-6 and ferritin had a tend to associate with death (*P* = 0.066 and *P* = 0.056, respectively). The serum CCL2 level was significantly higher in the death group (1655.7 [893.7–4642.8] pg/mL) than in the survival group (608.5 [193.2–3075.1] pg/mL) (*P* = 0.007). However, no significant differences were noted in serum CXCL9, CXCL 10, CXCL11, and CCL17 levels between the two groups. The P/F ratio was significantly lower in the death group (289.3 [215.8–320.5] torr) than in the survival group (382.1 [222.1–495.7] torr) (*P* = 0.002). The respiratory function test results showed no significant differences in %VC and %DLco between the groups. In chest HRCT scoring, the total GGO score was higher in the death group (15.7 [8.3–18.7]) than in the survival group (9.3 [3.7–18.3]) (*P* = 0.035), and there was no difference in the total fibrosis score between the two groups. All the patients received treatment after admission to our hospital. In terms of treatment, no differences were observed in the dose of PDN, CSA, and total IVCY, and the frequency of methylprednisolone pulse therapy between the two groups, but intravenous immunoglobulin was used more frequently in the death group (*P* = 0.016).

**Table 1:** Comparison of clinical characteristics, chemokine levels, and contents of treatment between in survivors and dead.

Characteristics	Dead (n = 6)	Alive (n = 24)	*P*
Age, years	64.5 (50–78)	59 (24–84)	0.604
Female, n (%)	3 (50)	16 (67)	0.641
CADM, n (%)	6 (100)	13 (54)	0.196
A/SIP, n (%)	6 (100)	16 (67)	0.155
Disease duration, months	2.2 (1–3.1)	1.6 (0.1–32.9)	0.586
Positive anti-MDA5ab, n (%)	4 (67)	4 (17)	0.029*
Positive anti-ARS ab, n (%)	1 (17)	10 (45)^a^	0.062
CK, IU/l	213 (43–721)	111 (23–5330)	0.678
ALD, IU/l	10.7 (5.2–11.6)	8.0 (3.1–94.6)	0.641
LDH, IU/l	376.5 (272–509)	324 (153–711)	0.421
CRP, mg/dl	0.91 (0.48–2.85)	0.41 (0.03–14.5)	0.422
KL-6, U/ml	1780 (501–3898)	626 (137–4508)	0.066
Ferritin, ng/ml	1056.5 (123–1611)	235.2 (15–23272.5)^a^	0.056
CXCL9, pg/mL	1968.8 (360.7–4053.3)	474.2 (125.3–9559.5)	0.272
CXCL10, pg/ml	275.3 (222.7–1470.8)	209.8 (17.4–1415.6)	0.17
CXCL11, pg/mL	261.9 (45.7–1930.1)	135.1 (2.19–636.4)	0.312
CCL2, pg/mL	1655.7 (893.7–4642.8)	608.5 (193.2–3075.1)	0.007*
CCL17, pg/mL	503.5 (138.0–1421.2)	574.4 (156.4–12747.4)	0.337
P/F ration, torr	289.3 (215.8–320.5)	382.1 (222.1–495.7)	0.002*
%VC, %	80.5 (48.1–103.4)^b^	82.6 (52.1–120.6)^c^	0.776
%DLco, %	39.9 (15.3–80.2)^b^	47.9 (11.74–104.8)^c^	0.903
Total GGO score	15.7 (8.3–18.7)	9.3 (3.7–18.3)	0.035*
Total fibosis score	4.85 (1.3–5)	3.3 (2–5)	0.229
PDN (n = 30), mg/day	57.5 (35–80)	50 (20–80)	0.297
CSA (n = 18), mg/day	225 (200–325)^d^	225 (175–325)^e^	0.735
TAC (n = 12), mg/day		4.5 (2.5–12.0)^e^	
MPDN pulse, n (%)	3 (50)	3 (13)	0.075
Total IVCY (n = 17), mg	2050 (500–4900)^d^	1300 (200–7000)^f^	0.513
IVIG, n (%)	4 (67)	3 (13)	0.016*
Dead due to other factors (n = 5) Four died due to infections and 1 died due to TTP			

The laboratory markers are presented as the median (interquartle range). The *P*-values were estimated using Fisher’s exact test or Mann-Whitney U- test. *P < 0.05. Dead: dead due to interstitial pneumonia; CADM: clinically amyopathic dermatomyositis; A/SIP: acute/subacute interstitial pneumonia; MDA5: melanoma differentiation-associated gene 5; ARS: aminoacyl-tRNA synthetase; ab: antibody; CK: creatine kinase; ALD: aldolase; LDH: lactate dehydrogenase; CRP: C-reactive protein; KL-6: Krebs von den Lungen-6; CXCL: C-X-C motif chemokine ligand; CCL: C-C motif chemokine ligand; P/F: PaO_2_/FiO_2_; VC: Vital capacity; DLco: diffusion capacity of the lung for carbon monoxide; GGO: ground-glass opacity; PDN: prednisolone; CSA: cyclosporine; TAC: tacrolimus; MPDN pulse: methylprednisolone pulse therapy; IVCY: intravenous pulse cyclophosphamide; IVIg: intravenous immunoglobulin; TTP: thrombotic thrombocytopenic purpura. ^a^Number of subjects, n = 23. ^b^Number of subjects, n = 4. ^c^Number of subjects, n = 19. ^d^Number of subjects, n = 6. ^e^Number of subjects, n = 12. ^f^Number of subjects, n = 11.

### Multivariate analysis of baseline values on longitudinal changes in the non-survivors

To examine the factors associated with death due to IP, logistic regression analysis by backward elimination was performed using factors such as age, sex, disease duration, positive anti-MDA5 antibody, CCL2, P/F ratio, and total GGO score, which were associated with death in the univariate analysis. CCL2 and P/F ratio were found to significantly relate to death (*P* = 0.018 and 0.009, respectively).

There were no correlations between serum CCL2 level and serum KL-6 level or serum ferritin level.

### Cut-off values of initial serum CCL2 levels, P/F ratio, KL-6, and ferritin and survival rates

**Figure 1:**
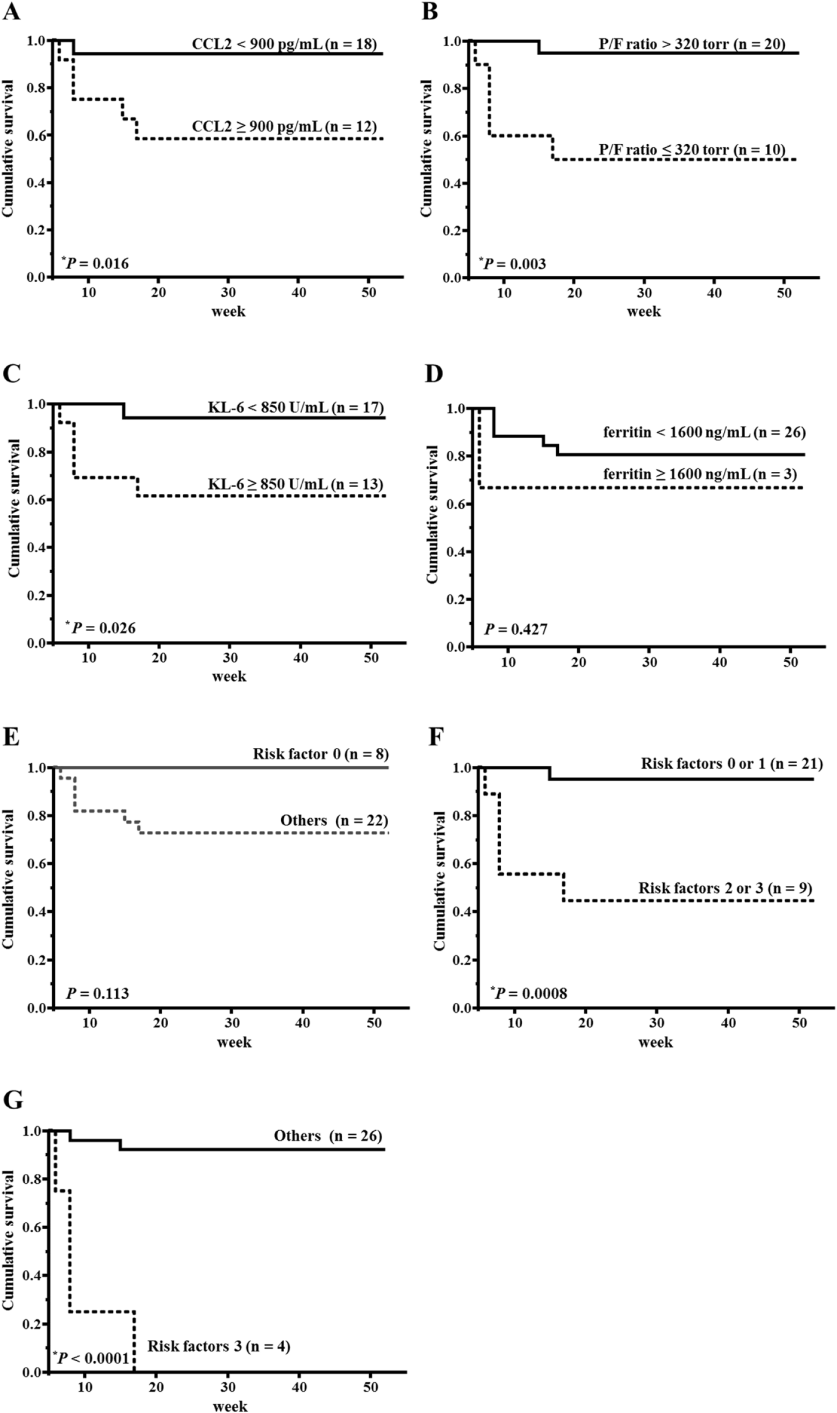
Survival curve of patients based on their initial serum CCL2 levels, P/F ratio, KL-6, and ferritin. (**A**) Survival curve of patients based on their initial serum CCL2 levels (solid line: <900 pg/mL; dashed line: ≥900 pg/mL). (**B**) Survival curve of patients based on their initial P/F ratio (solid line: >320 torr; dashed line: ≤320 torr). (**C**) Survival curve of patients based on their initial KL-6 (solid line: <850 mmHg; dashed line: ≥850 mmHg). (**D**) Survival curve of patients based on their initial ferritin (solid line: <1600 ng/mL; dashed line: ≥1600 ng/mL). (**E**) Survival curve of patients based on their initial risk factors (solid line: 0 risk factors; dashed line: others). (**F**) Survival curve of patients based on their initial risk factors (solid line: 0 or 1 risk factor; dashed line: 2 or 3 risk factors). (**G**) Survival curve of patients based on their initial risk factors (solid line: others; dashed line: 3 risk factors). CCL: C-C motif chemokine ligand; P/F: PaO_2_/FiO_2_. Survival rates were calculated by the Kaplan-Meier method and compared by log-rank test. *P < 0.05.

To determine the cut-off points effective as poor prognostic factors of DM-IP, ROC curve analysis was carried out on initial serum CCL2 levels, P/F ratio, KL-6, and ferritin. The value that maximized the area under the ROC curve was 894 pg/mL for initial serum CCL2 level (sensitivity: 100%, specificity: 70.8%), 321 torr for P/F ratio (sensitivity: 100%, specificity: 79.2%), 864 U/mL for KL-6 (sensitivity: 83.3%, specificity: 54.2%), and 1611 ng/mL for ferritin (sensitivity: 100%, specificity: 8.7%). From these results, a serum CCL2 level of ≥900 pg/mL, a P/F ratio of ≤320 torr, a serum KL-6 level of ≥850 U/mL, and a serum ferritin level of ≥1600 ng/mL were determined as cut-off values for a poor prognosis. The patients were then divided into two groups on the basis of these cut-off values, and Kaplan-Meier survival curves were plotted (Fig. 1A,B,C,D). The survival rate after 52 weeks was significantly lower in patients with a level of CCL2 of ≥900 pg/mL (survival rate: 58.3%) than in those with <900 pg/mL (94.4%) (*P* = 0.016). The survival rate was also significantly lower in patients with a P/F ratio of ≤320 torr (survival rate: 50%) than in those with >320 torr (95%) (*P* = 0.003). The survival rate was also significantly lower in patients with a KL-6 of ≥850 U/mL (survival rate: 61.5%) than in those with <850 U/mL (94.1%) (*P* = 0.026). The survival rate did not significant change between ≥1600 U/mL and <1600 U/mL. Figure 1E,F and G show Kaplan-Meier survival curves based on the number of prognostic factors: level of CCL2 of ≥900 pg/mL, P/F ratio of ≤320 torr, and KL-6 of ≥850 U/mL. There was no significant change between no risk factor and the others. The survival rate was significantly lower in patients with 2 or 3 risk factors (survival rate: 44.4%) than in those with 0 or 1 factor (95.2%) (*P* = 0.0008). The survival rate was significantly lower in patients with 3 risk factors (survival rate: 0%) than in the others (92.3%) (*P* < 0.0001), and all patients who had 3 risk factors died within 17 weeks.

### Changes in and Comparison of serum chemokine levels of DM-IP initially and at 2weeks after treatment between survivors and non-survivors

**Figure 2:**
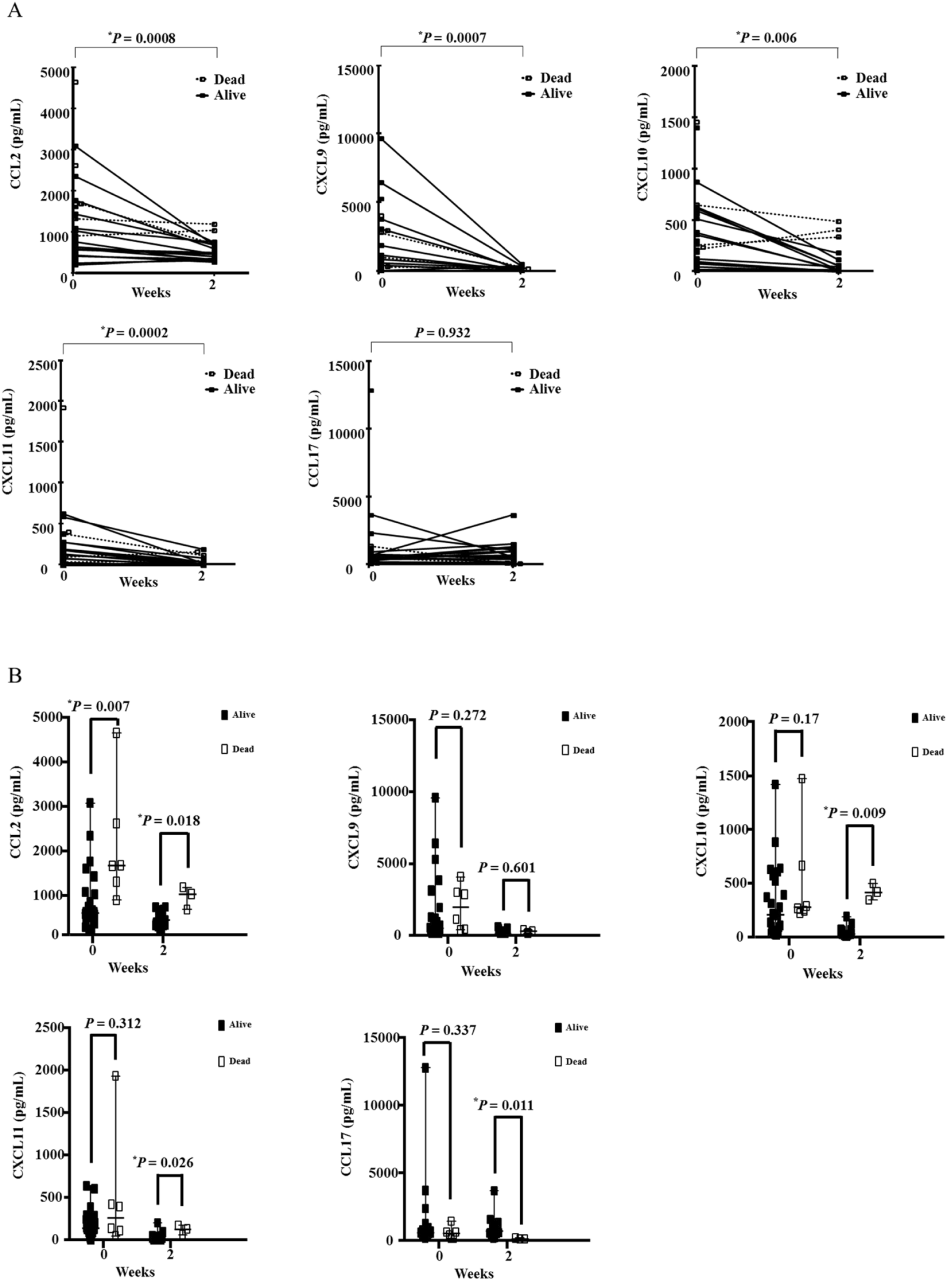
Changes in and Comparison of serum chemokine levels of DM-IP initially and at 2 weeks after treatment between survivors and non-survivors. (**A**) The time series of each chemokine; (**B**) The comparison of serum chemokine levels initially and at 2 weeks after treatment between the survival group and the death group; CCL: C-C motif chemokine ligand; CXCL: C-X-C motif chemokine ligand; closed square: alive patients (Alive); open square: patients dead due to interstitial pneumonia (Dead). The *P* value was estimated by Wilcoxon’s rank sum test. *P < 0.05.

Figure 2A shows the time series of each chemokine. Serum CCL2, CXCL9, CXCL10, and CXCL11 levels were significantly lower at 2 weeks after treatment initiation than before treatment (0–2 weeks: *P* = 0.0008, 0.0007, 0.006, and 0.0002, respectively; There was no apparent change in serum CCL17 level throughout the treatment course. Figure 2B shows the comparison of serum chemokine levels initially and at 2 weeks after treatment between the survival group and the death group. Serum CCL2 levels at both times were significantly higher in the death group (1655.7 [893.7–4642.8] and 1030.7 [684–1182.6] pg/mL, respectively) than in the survival group (608.5 [193.2–3075.1] and 450.6 [262.7–745.7] pg/mL, respectively) (*P* = 0.007 and 0.018, respectively). At 2 weeks after treatment initiation, the respective serum CXCL 10 levels in the death group (416.2 [345.9–498.3] pg/mL) were significantly higher than those in the survival group (26.6 [10.2–189.8] pg/mL) (*P* = 0.009). At 2 weeks after treatment initiation, the respective serum CXCL 11 levels in the death group (125.5 [60.3–169.1] pg/mL) were also significantly higher than those in the survival group (2.19 [2.19–198.2] pg/mL) (*P* = 0.026). However, no difference was observed at treatment initiation. The serum CCL17 level at 2 weeks after treatment initiation was higher in the survival group (718.9 [172.8–3660.1] pg/mL) than in the death group (93.92 [93.92–172.8] pg/mL) (*P* = 0.011), but there were no differences at the other times.

### Comparison of the change ratio of chemokine levels between initially and at 2 weeks after treatment

The change ratio of chemokine levels between before treatment and 2 weeks after initial treatment was examined between the survival group and the death group (Supplementary Fig. S1). The change ratio of chemokine levels between initially and at 2 weeks after treatment were calculated using the following formula: (chemokine level at 2 weeks after treatment initiation – initial chemokine level)/initial chemokine level. The change ratio of serum CXCL10 and CXCL 11 levels were significantly higher in the death group (*P* = 0.033 and 0.028, respectively). The change ratio of serum CCL17 level was significantly higher in the survival group (*P* = 0.024). No significant differences were observed in CCL2 and CXCL9.

### Cut-off values of serum chemokine levels for determining poor prognostic factors of DM-IP

To determine the cut-off points effective as poor prognostic factors of DM-IP, we performed ROC curve analysis on the serum chemokine levels (CCL2, CXCL10, and CXCL11) at 2 weeks after treatment initiation. At 2 weeks after treatment initiation, the values of these levels that maximized the area under the ROC curve were 684 pg/mL (sensitivity: 100%, specificity: 86.7%), 346 pg/mL (sensitivity: 100%, specificity: 100%), and 60 pg/mL (sensitivity: 100%, specificity: 86.7%), respectively.

### Comparison of each chemokine between anti-MDA5 antibody-positive and -negative cases

**Figure 3:**
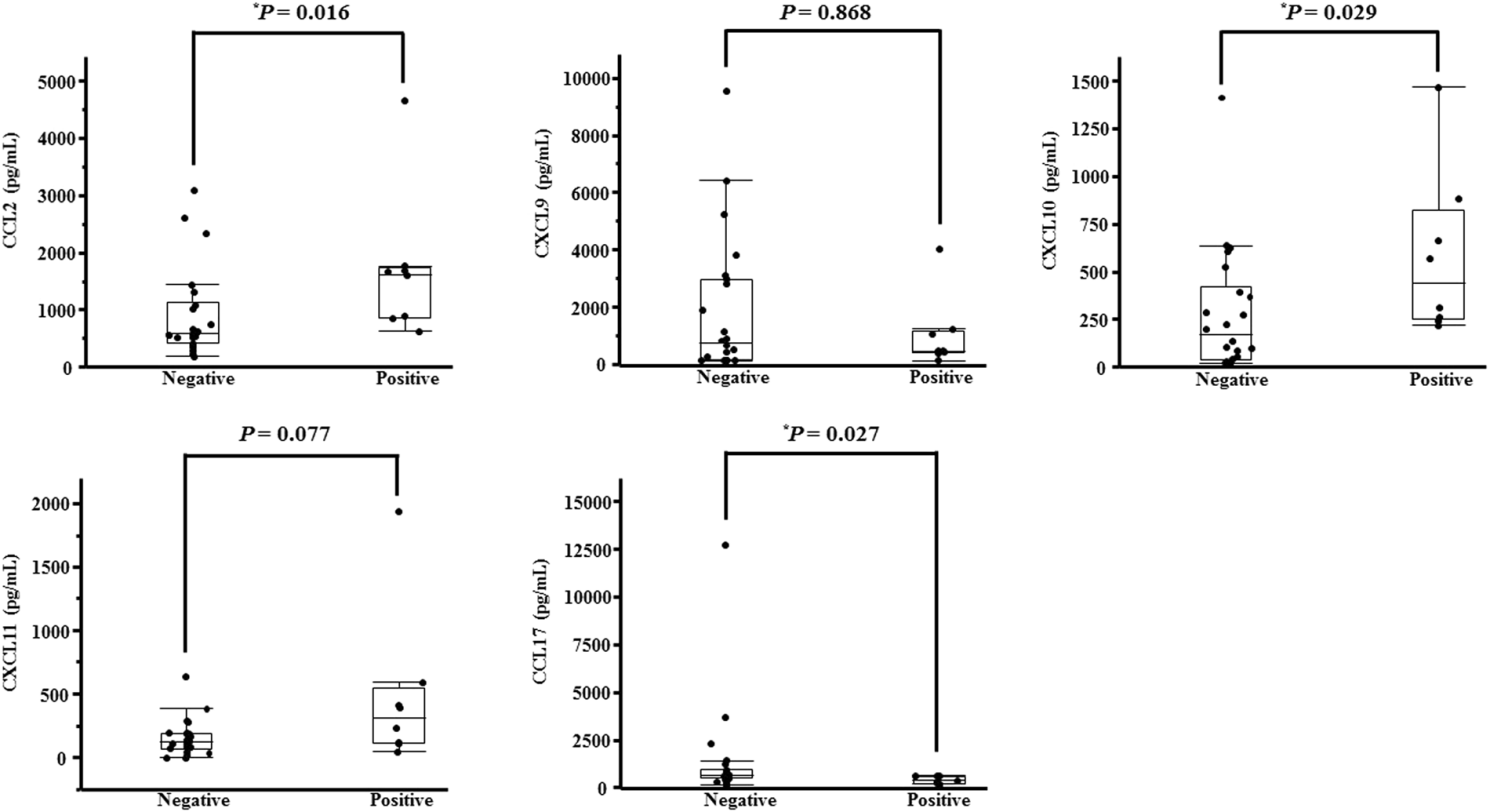
Comparison of each of the chemokine levels between anti-MDA5 antibody-positive and -negative cases. CCL: C-C motif chemokine ligand; CXCL: C-X-C motif chemokine ligand; negative: anti-MDA5 antibody-negative patients; positive: anti-MDA5 antibody-positive patients. The *P* value was estimated by the Mann-Whitney U-test. *P < 0.05.

A comparison of each of the chemokine levels between anti-MDA5 antibody-positive and -negative cases is shown in Fig. 3. The serum CCL2 level was significantly higher in the anti-MDA5-positive cases (1625.9 [628.7–4642.8] pg/mL) than in the anti-MDA5-negative cases (584 [193.2–3075.1] pg/mL) (*P* = 0.016). The serum CXCL10 level was also significantly higher in the anti-MDA5-positive cases (440.1 [220.7–1470.8] pg/mL) than in the anti-MDA5-negative cases (167.3 [17.4–415.6] pg/mL) (*P* = 0.029). In contrast, the serum CCL17 level was significantly higher in the anti-MDA5-negative cases (627.8 [138–12747.4] pg/mL) than in the anti-MDA5-positive cases (354.8 [156.4–615.8] pg/mL) (*P* = 0.027). Other chemokines were not significantly different between these cases.

## Discussion

We examined the association between biomarkers including serum chemokines and disease activity or prognosis of DM-IP. Serum CCL2 level, P/F ratio, total GGO score, and anti-MDA5 antibody positivity before treatment were all significantly higher in the survival group than in the death group. Serum CCL2 and P/F ratio before treatment independently related to death. CCL2 and Th1 chemokines (CXCL9, CXCL10, and CXCL11) significantly decreased after treatment, but there was no change in the Th2 chemokine (CCL17). The change ratio of serum CXCL10 and CXCL11 levels were higher in the death group, whereas that of serum CCL17 level was lower. Serum CCL2, CXCL10, and CXCL11 levels at 2 weeks after treatment initiation were significantly higher in the death group, whereas the serum CCL17 level at 2 weeks after treatment initiation was lower. In the anti-MDA5 antibody-positive cases, serum CCL2 and CXCL10 levels were higher, and the serum CCL17 level was lower, compared with those in the negative cases.

CCL2 is a classic chemokine that relates to activated macrophages, which are related to the pathophysiology of DM-IP. In the autopsy of a patient with DM-IP, ferritin-phagocytosed macrophages were observed diffusely in the lung, liver, spleen, and bone marrow^41^. Activated macrophages were also found in bronchoalveolar lavage fluid from patients with DM-IP^42^. Ferritin, tumor necrosis factor-α, and IL-18 are related to the activation of macrophages, and these biomarkers correlate with the disease activity of DM-IP^18–20, 28, 31, 32^. The serum CCL2 level was significantly higher in patients with DM-IP than in those with DM without IP^33^. In the present study, serum CCL2 levels before and after treatment were related to the prognosis of DM-IP. This suggests that serum CCL2 level before and after treatment initiation could be a possible marker for disease activity and prognosis in DM-IP.

Th1-type immune response plays an important role in the pathophysiology of DM-IP. Kurasawa *et al*. reported that activated Th1-type pulmonary T cells and CD8+CD25+ T cells are associated with the development of corticosteroid-resistant DM-IP^43^. In DM-IP, lymphocytes infiltrating in lung tissue are mainly CD8+ T and Th1 cells^44, 45^. Richards *et al*. reported that serum CXCL9 and CXCL10 levels were higher in patients with IP with anti-Jo-1 antibody than in those with idiopathic pulmonary fibrosis^46^. Gono *et al*. reported that the serum CXCL10 level related to the disease activity of PM/DM-IP^28^. In our study, serum CXCL10 and CXCL11 levels at 2 weeks after treatment initiation were significantly higher in the death group and the change ratio of serum CXCL10 and CXCL11 levels were higher in the death group. This suggests that serum CXCL10 and CXCL11 levels could be possible markers for prognosis during the treatment course. In contrast, the serum level and the change ratio of CCL17 as the Th2-chemokine were lower in the death group. This suggests that Th2-type immune responses may be suppressed in DM-IP.

Anti-MDA5 antibody is reported to be a possible prognostic marker in DM-IP^16, 17^. It was reported that the IL-4/interferon-γ ratio is lower in anti-MDA5 antibody cases than in anti-ARS antibody-positive cases^28^. In the present study, serum CCL2 and CXCL10 levels were significantly higher, and the serum CCL17 level was lower, in anti-MDA5 antibody-positive group. These findings suggest that macrophage activation was more active and that the Th1/Th2 balance shifted to a predominance of Th1-type immune responses in the anti-MDA5 antibody-positive group as compared with the anti-MDA5 antibody-negative group.

Positive anti-MDA5 antibody, high serum ferritin level, and high serum IL-6 level before treatment were previously reported as prognostic factors of DM-IP^16–21, 29^. Although serum IL-6 was not examined in this study, the anti-MDA5 antibody-positive rate was higher in the death group, similar to findings in previous reports. However, there was no significant correlation between the serum ferritin level and prognosis in the present study. We speculate that intensive combination therapy with PDN and calcineurin inhibitors and/or IVCY was administered early after the diagnosis of DM-IP in some patients with high serum ferritin levels.

## Conclusion

We showed that serum levels of chemokines, especially that of CCL2, may be possible biomarkers of disease activity and prognosis in patients with DM-IP. Furthermore, serum CCL2 levels and P/F ratio may also be useful when deciding initial treatment. This study was retrospective and involved a small number of patients in only one institution. Further investigations are thus needed to clarify the usefulness of serum chemokines as biomarkers of disease progression, activity, and prognosis of DM-IP.

## Electronic supplementary material

Supplementary Information, S1
