# Supplementary material for: Chemokine profiles of interstitial pneumonia in patients with dermatomyositis: a case control study
Source: Sci Rep. 2017 May 9;7:1635. doi: 10.1038/s41598-017-01685-5 (PMC5431618; doi:10.1038/s41598-017-01685-5)
Supplement: Supplementary file 1 — Supplementary Information, S1 [file 41598_2017_1685_MOESM1_ESM.pdf]

Supplementary Information for:

Chemokine profiles of interstitial pneumonia in patients with dermatomyositis: a case control study

Katsuhiro Oda, MD[1], Takuya Kotani, MD, PhD[1], Tohru Takeuchi, MD, PhD[1], Takaaki Ishida, MD[1], Takeshi Shoda, MD[1], Kentaro Isoda, MD, PhD[1], Shuzo Yoshida, MD, PhD[1],Yasuichiro Nishimura, PhD[2], and Shigeki Makino, MD, PhD[1]

[1]Department of Internal Medicine (I), Osaka Medical College, Takatsuki, Osaka, Japan

[2]Department of Mathematics, Osaka Medical College, Takatsuki, Osaka, Japan

Correspondence and reprint requests to: Takuya Kotani, MD, PhD,

Department of Internal Medicine (I), Osaka Medical College, Daigaku-Machi 2-7, Takatsuki, Osaka 569-8686, Japan

Tel.: +81-72-683-1221; Fax: +81-72-683-1801

E-mail: in1242@osaka-med.ac.jp

This supplement contains:

Supplementary Figure S1

Supplementary Fig. S1. Comparison of the change ratio of chemokine levels initially and at 2 weeks after treatment.

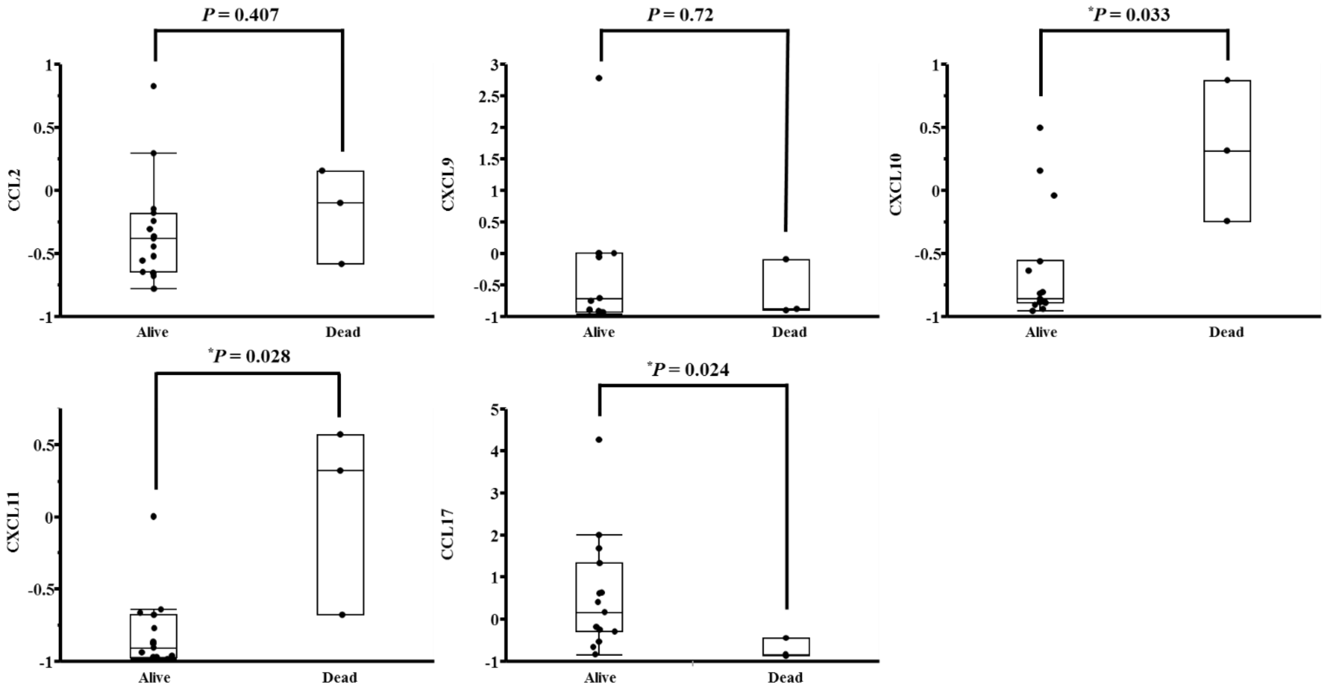

CCL: C-C motif chemokine ligand; CXCL: C-X-C motif chemokine ligand. The P value was estimated by the Mann-Whitney U-test. $^*P < 0.05$.
